# The Story of "Sister Olive"

**Published:** 1887-01-08

**Authors:** A. Baster

**Affiliations:** Lady Superintendent. Royal Berks Hospital, Reading


I am pleased to see Miss Wood's objection to the story "Sister Olive." I should have written to you myself a fortnight ago on the subject, but for a pressure of work at that time. My objection to the interview between Sister Olive and Dr. Grant in the house-physician's room had not a shadow of imputation or suspicion against them, but I think the higher their reputation the more harm will such a precedent do.

That hospital was singularly fortunate to have a resident medical officer of unblemished reputation for five years; so many changes and chances are usually taking place in a hospital, now that strict rules are needed more than formerly. Happy must any hospital be which has not in its annals a record of some incidents that prove it is undesirable for the nursing staff to visit the resident medical officers in their own rooms. I am disappointed to find you think The Hospital needs the addition of a story which seems to me more suited for the pages of the *Family Herald.*

A. Baster, Lady Superintendent.

Royal Berks Hospital, Reading.

